# New Benzo[*c*]phenanthridine and Benzenoid Derivatives, and Other Constituents from *Zanthoxylum ailanthoides*: Effects on Neutrophil Pro-Inflammatory Responses

**DOI:** 10.3390/ijms141122395

**Published:** 2013-11-13

**Authors:** Ching-Yi Chung, Tsong-Long Hwang, Liang-Mou Kuo, Wen-Lung Kuo, Ming-Jen Cheng, Yi-Hsiu Wu, Ping-Jyun Sung, Mei-Ing Chung, Jih-Jung Chen

**Affiliations:** 1 Faculty of Pharmacy, College of Pharmacy, Kaohsiung Medical University, Kaohsiung 807, Taiwan; E-Mail: dream6637@hotmail.com; 2 Graduate Institute of Natural Products, College of Medicine, Chang Gung University, Taoyuan 333, Taiwan; E-Mail: htl@mail.cgu.edu.tw (T.-L.H.); modemtw@yahoo.com (Y.-H.W.); 3 Graduate Institute of Clinical Medical Sciences, College of Medicine, Chang Gung University, Taoyuan 333, Taiwan; E-Mail: kuo33410@yahoo.com.tw; 4 Department of General Surgery, Chang Gung Memorial Hospital at Chia-Yi, Chia-Yi 613, Taiwan; 5 Chung-Jen College of Nursing, Health Science and Management, Chiayi 600, Taiwan; E-Mail: m049@cjc.edu.tw; 6 Bioresource Collection and Research Center (BCRC), Food Industry Research and Development Institute (FIRDI), Hsinchu 300, Taiwan; E-Mail: cmj0404@gmail.com; 7 National Museum of Marine Biology and Aquarium, Pingtung 944, Taiwan; E-Mail: pjsung@nmmba.gov.tw; 8 Department of Pharmacy & Graduate Institute of Pharmaceutical Technology, Tajen University, Pingtung 907, Taiwan

**Keywords:** *Zanthoxylum ailanthoides*, Rutaceae, benzo[*c*]phenanthridine, benzenoid, anti-inflammatory activity

## Abstract

A new benzo[*c*]phenanthridine, oxynorchelerythrine (**1**), and two new benzenoid derivatives, methyl 4-(2-hydroxy-4-methoxy-3-methyl-4-oxobutoxy)benzoate (**2**) and (*E*)-methyl 4-(4-((*Z*)-3-methoxy-3-oxoprop-1-enyl)phenoxy)-2-methylbut-2-enoate (**3**), have been isolated from the twigs of *Zanthoxylum ailanthoides*, together with 11 known compounds (**4**–**14**). The structures of these new compounds were determined through spectroscopic and MS analyses. Among the isolated compounds, decarine (**4**), (−)-syringaresinol (**6**), (+)-episesamin (**8**), glaberide I (**9**), (−)-dihydrocubebin (**10**), and xanthyletin (**11**) exhibited potent inhibition (IC_50_ values ≤ 4.79 μg/mL) of superoxide anion generation by human nutrophils in response to *N*-formyl-l-methionyl-l-leucyl-l-phenylalanine/cytochalasin B (fMLP/CB). Compounds **4**, **8**, and **11** also inhibited fMLP/CB-induced elastase release with IC_50_ values ≤ 5.48 μg/mL.

## 1. Introduction

*Zanthoxylum ailanthoides* Sieb. & Zucc. (Rutaceae) is a medium-to-large-sized tree, found at low altitude in forests of China, Japan, Korea, Philippines, and Taiwan [1]. Various benzo[*c*]phenanthridines, coumarins, lignans, flavonoids, quinolines, benzenoids, and triterpenoids are widely distributed in this plant [2–12]. Many of these compounds exhibit anti-platelet aggregation [10], anti-HIV [11], and anti-inflammatory [12] activities. Granule proteases (e.g., elastase, cathepsin G, and proteinase-3) and reactive oxygen species (ROS) (e.g., superoxide anion (O_2_^•−^) and hydrogen peroxide) produced by human neutrophils are involved in the pathogenesis of a variety of inflammatory diseases.

**Figure 1:**
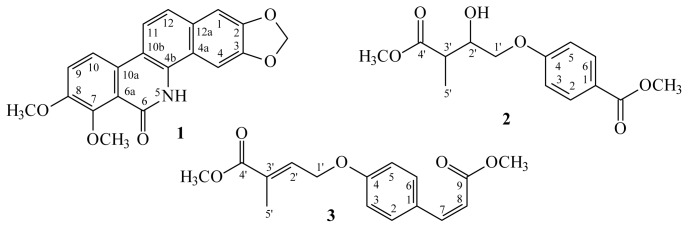
The chemical structures of new compounds **1**–**3** isolated from *Zanthoxylum ailanthoides*.

**Figure 2:**
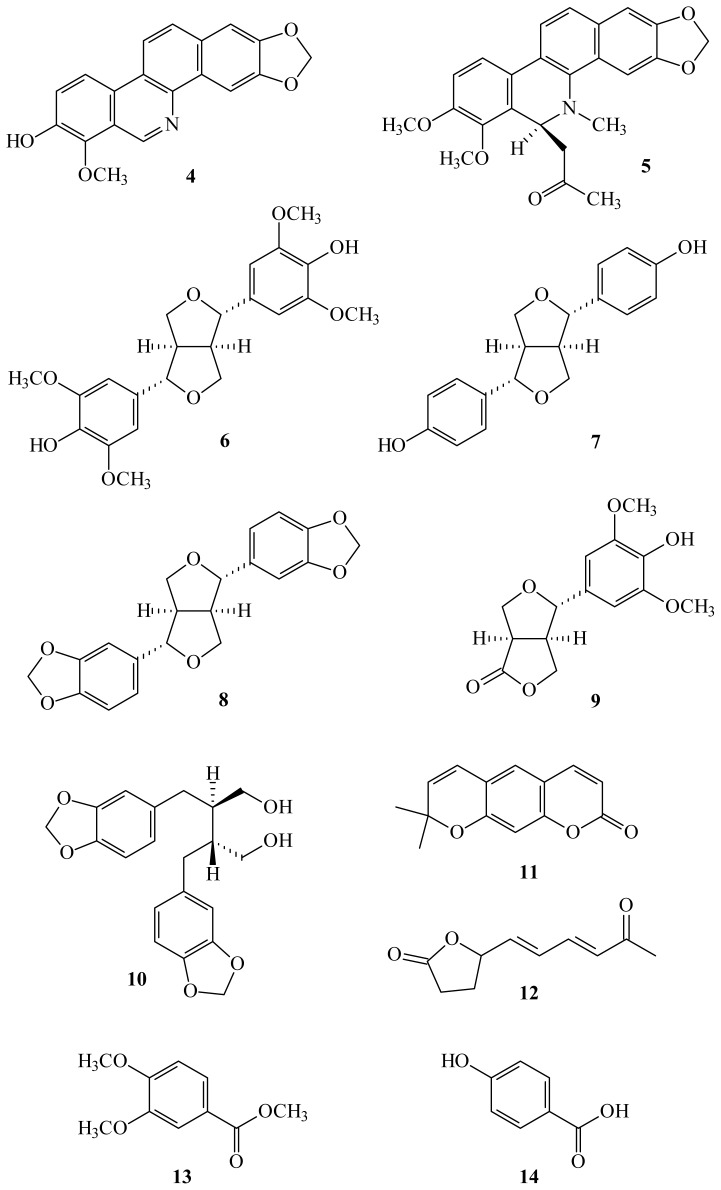
The chemical structures of known compounds **4**–**14** isolated from *Zanthoxylum ailanthoides*.

In our studies of Formosan plants for *in vitro* anti-inflammatory activity, *Z. ailanthoides* was found to be an active species. The MeOH extract of the twigs of *Z. ailanthoides* showed potent inhibitory effects on superoxide anion generation and elastase release by human neutrophils in response to formyl-l-methionyl-l-leucyl-l-phenylalanine/cytochalasin B (fMLP/CB). Figure 1 illustrates the structures of a new benzo[*c*]phenanthridine, oxynorchelerythrine (**1**) and two new benzenoid derivatives, methyl 4-(2-hydroxy-4-methoxy-3-methyl-4-oxobutoxy)benzoate (**2**) and (*E*)-methyl 4-(4-((*Z*)-3-methoxy-3-oxoprop-1-enyl)phenoxy)-2-methylbut-2-enoate (**3**). Eleven known compounds (**4**–**14**), have been isolated and identified from the twigs of *Z. ailanthoides* and their structures are depicted in Figure 2.

This paper describes the structural elucidation of the compounds numbered **1** through **3**, and the inhibitory activities of all isolates on superoxide generation and elastase release by neutrophils.

## 2. Results and Discussion

**Figure 3:**
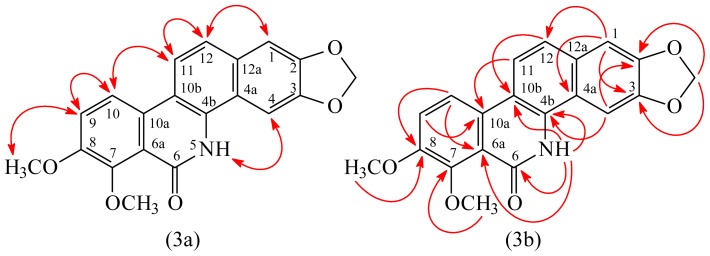
Key NOESY (**3a**) and HMBC (**3b**) correlations of **1**.

Oxynorchelerythrine (**1**) was isolated as a white amorphous powder. Its molecular formula, C_20_H_15_NO_5_, was determined on the basis of the *quasi*-molecular ion at *m/z* 372.0846 ([M + Na]^+^, calcd for C_20_H_15_NO_5_Na: 372.0848) in the HR-ESI-MS spectrum (positive-ion mode) (Figures S1 and S2) and was supported by the ^1^H-, ^13^C-, and DEPT NMR data. The UV absorptions of **1** at 236, 281, and 286 nm were similar to those of oxychelerythrine [13], and suggested the presence of a 2,3,7,8-tetraoxygenated benzo[*c*]phenanthridin-6-one skeleton. The presence of carbonyl group was revealed by the band at 1644 cm^−1^ in the IR spectrum, which was confirmed by the resonance at δ_C_ 162.4 in the ^13^C-NMR spectrum. The IR of **1** also showed the NH absorption at 3218 cm^−1^ and the methylenedioxy bands at 1040, 938 cm^−1^. The ^1^H-NMR spectrum of **1** showed the resonances for six aromatic protons [δ_H_ 7.20 (1H, s, H-1), 7.43 (1H, br s, H-4), 7.46 (1H, d, *J* = 9.0 Hz, H-9), 7.51 (1H, br d, *J* = 9.0 Hz, H-12), 8.02 (1H, d, *J* = 9.0 Hz, H-11), 8.09 (1H, d, *J* = 9.0 Hz, H-10), two methoxy groups [δ_H_ 4.01 (3H, s, OMe-8), 4.05 (3H, s, OMe-7)], a methylenedioxy group [δ_H_ 6.13 (2H, s, OCH_2_O-2,3)], and an NH group [δ_H_ 9.14 (1H, br s, D_2_O exchangeable, NH)]. Comparison of the ^1^H- and ^13^C-NMR data (Table 1) (Figures S3 and S4) of **1** with those of oxychelerythrine [14] suggested that their structures are closely related, except that the NH group (δ_H_ 9.14) of **1** replaced the N-Me group [δ_H_ 3.89 (3H, s)] of oxychelerythrine [14]. This was supported by HMBC correlations between NH (δ_H_ 9.14) and C-4b (δ_C_ 135.6), C-6 (δ_C_ 162.4), C-6a (δ_C_ 119.7), and C-10b (δ_C_ 128.9) and NOESY correlations between NH (δ_H_ 9.14) and H-4 (δ_H_ 7.43). The full assignment of ^1^H- and ^13^C-NMR resonances was supported by ^1^H–^1^H COSY, DEPT, HSQC, NOESY (Figure 3), and HMBC (Figure 3) spectral analyses. On the basis of the above data, the structure of **1** was elucidated as oxynorchelerythrine.

**Figure 4:**
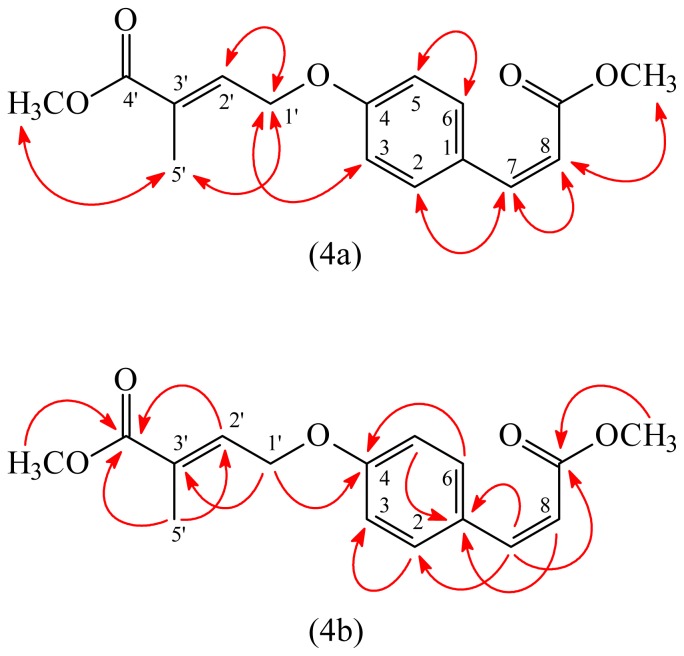
Key NOESY (**4a**) and HMBC (**4b**) correlations of **3**.

Methyl 4-(2-hydroxy-4-methoxy-3-methyl-4-oxobutoxy)benzoate (**2**) was isolated as colorless oil. The ESI-MS afford the *quasi*-molecular ion [M + Na]^+^ at *m/z* 305 (Figure S5), implying a molecular formula of C_14_H_18_O_6_Na, which was confirmed by the HR-ESI-MS (*m/z* 305.1003 [M + Na]^+^, calcd 305.1001) (Figure S6). The presence of two carbonyl groups was revealed by the bands at 1714 and 1728 cm^−1^ in the IR spectrum, which was confirmed by the resonances at δ 166.7 and 175.7 in the ^13^C-NMR spectrum. The ^1^H- and ^13^C-NMR data (Table 1) (Figures S7 and S8) of **2** were similar to those of methyl 4-hydroxybenzoate [15], except that the 2-hydroxy-4-methoxy-3-methyl-4-oxobutoxy group [δ_H_ 1.30 (3H, d, *J* = 7.0 Hz, H-5′), 2.88 (1H, *m*, H-3′), 3.09 (1H, br s, D_2_O exchangeable, OH-2′), 3.74 (3H, s, OMe-4′), 4.11 (3H, *m*, H_2_-1′ and H-2′); δ_C_ 14.1 (C-5′), 42.0 (C-3′), 52.0 (OMe-4′), 69.8 (C-1′), 71.8 (C-2′), 175.7 (C-4′)] at C-4 of **2** replaced the 4-hydroxy group [δ_H_ 6.58 (1H, s)] of methyl 4-hydroxybenzoate [15]. This was supported by HMBC correlations between H-1′ (δ_H_ 4.11) and C-4 (δ_C_ 162.1), C-2′ (δ_C_ 71.8), and C-3′ (δ_C_ 42.0) and NOESY correlations between H-1′ (δ_H_ 4.11) and H-3/5 (δ_H_ 6.93), H-3′ (δ_H_ 2.88), and H-5′ (δ_H_ 1.30). According to the above data, the structure of **2** was elucidated as methyl 4-(2-hydroxy-4-methoxy-3-methyl-4-oxobutoxy)benzoate (**2**). This was further confirmed by the ^1^H–^1^H-COSY, NOESY (Table 1), DEPT, HSQC, and HMBC (Table 1) techniques.

(*E*)-Methyl 4-(4-((*Z*)-3-methoxy-3-oxoprop-1-enyl)phenoxy)-2-methylbut-2-enoate (**3**) was isolated as an amorphous powder. The molecular formula C_16_H_18_O_5_ was deduced from a sodium adduct ion at *m*/*z* 313.1055 [M + Na]^+^ (calcd 313.1052) in the HR-ESI mass spectrum (Figures S9 and S10). The presence of carbonyl groups was revealed by the band at 1715 cm^−1^ in the IR spectrum, which was confirmed by the resonances at δ 166.9 and 167.8 in the ^13^C-NMR spectrum. The ^1^H- and ^13^C-NMR data (Figures S11 and S12) of **3** were similar to those of (*E*)-methyl 4-(4-(3-hydroxypropyl)phenoxy)-2-methylbut-2-enoate [12], except that the (*Z*)-3-methoxy-3-oxoprop-1-enyl group [δ_H_ 3.73 (3H, s, OMe-9), 5.83 (1H, d, *J* = 12.4 Hz, H-8), 6.86 (1H, d, *J* = 12.4 Hz, H-7); δ_C_ 51.3 (OMe-9), 116.7 (C-8), 143.6 (C-7), 166.9 (C-9)] at C-1 of **3** replaced 3-hydroxypropyl group of (*E*)-methyl 4-(4-(3-hydroxypropyl)phenoxy)-2-methylbut-2-enoate [12]. This was supported by (i) the HMBC correlations (Figure 4) between between H-7 (δ_H_ 6.86) and C-1 (δ_C_ 127.3), C-2 (δ_C_ 132.2), C-6 (δ_C_ 132.2), and C-9 (δ_C_ 166.9); (ii) the NOESY correlation (Figure 4) between H-7 (δ_H_ 6.86) and H-2 (δ_H_ 7.69) and H-8 (δ_H_ 5.83); and (iii) the *cis*-coupling constant (*J* = 12.4 Hz) for H-7 and H-8 of **3**. The NOESY correlations between H-1′ (δ_H_ 4.69) and H-5′ (δ_H_ 1.93) suggested 2′*E*-configuration of **3**. The structure elucidation of **3** was supported by ^1^H–^1^H COSY and NOESY (Figure 4) experiments, and ^13^C NMR assignments were confirmed by DEPT, HSQC, and HMBC (Figure 4) techniques.

The known isolates were readily identified by a comparison of physical and spectroscopic data (UV, IR, ^1^H-NMR, [α]_D_, and MS) with corresponding authentic samples or literature values, and this included two benzo[*c*]phenanthridines, decarine (**4**) [16] and 6-acetonyldihydrochelerythrine (**5**) [17], five lignan derivatives, (−)-syringaresinol (**6**) [18], 5′,5″-didemethoxypinoresinol (**7**) [19], (+)-episesamin (**8**) [12], glaberide I (**9**) [20], and (−)-dihydrocubebin (**10**) [21], a coumarin, xanthyletin (**11**) [12], a lactone, lanyulactone (**12**) [22], and two benzenoids, methyl 3,4-dimethoxybenzoate (**13**) [23] and *p*-hydroxybenzoic acid (**14**) [24].

Human neutrophils are known to play an important roles in host defense against microorganisms and in pathogenesis of various diseases. In response to different stimuli, activated neutrophils secrete a series of cytotoxins, such as the superoxide anion radical (O_2_^•−^), a precursor to other reactive oxygen species (ROS), granule proteases, bioactive lipids, and neutrophil elastase, a major contributor to destruction of tissue in chronic inflammatory disease [25–28]. Suppression of the extensive or inappropriate activation of neutrophils by drugs has been proposed as a way to ameliorate inflammatory diseases. In this study, the effects on neutrophil pro-inflammatory responses of compounds isolated from the twigs of *Z. ailanthoides* were evaluated by suppressing fMLP/CB-induced superoxide radical anion (O_2_^•−^) generation and elastase release by human neutrophils. The inhibitory activity data on neutrophil pro-inflammatory responses are shown in Table 2. LY294002 (Sigma, St. Louis, MO, USA), a phosphatidylinositol-3-kinase inhibitior, was used as a positive control for O_2_^•−^ generation and elastase release, respectively [29,30]. From the results of our biological tests, the following conclusions can be drawn: (a) Compounds **4**, **6**, and **8**–**11** exhibited inhibitory activities (IC_50_ values ≤ 4.79 μg/mL) on human neutrophil O_2_^•−^ generation; (b) Compounds **4**, **5**, **8**, and **11** inhibited fMLP/CB-induced elastase release with IC_50_ values ≤ 7.12 μg/mL; (c) The benzo[*c*]phenanthridine derivative **4** {with 8-hydroxy group and double bond [N(5)=C(6)]} exhibited more effective inhibition than its analogues **4** (with NH, 6-oxo, and 8-methoxy groups) and **5** (with NMe, 6-acetonyl, and 8-methoxy groups) against fMLP-induced O_2_^•−^ generation and elastase release; (d) Glaberide I (**9**) (with a 6-oxo group) exhibited more effective inhibition than its analogue **6** (with a 4-hydroxy-3,5-dimethoxyphenyl group at C-6 against fMLP-induced O_2_^•−^ generation and elastase release; (e) Decarine (**4**) and (+)-episesamin (**8**) were the most effective among the isolated compounds, with IC_50_ values of 1.31 ± 0.18 and 1.42 ± 0.16 μg/mL, respectively, against fMLP-induced superoxide anion generation and elastase release.

**Figure 5:**
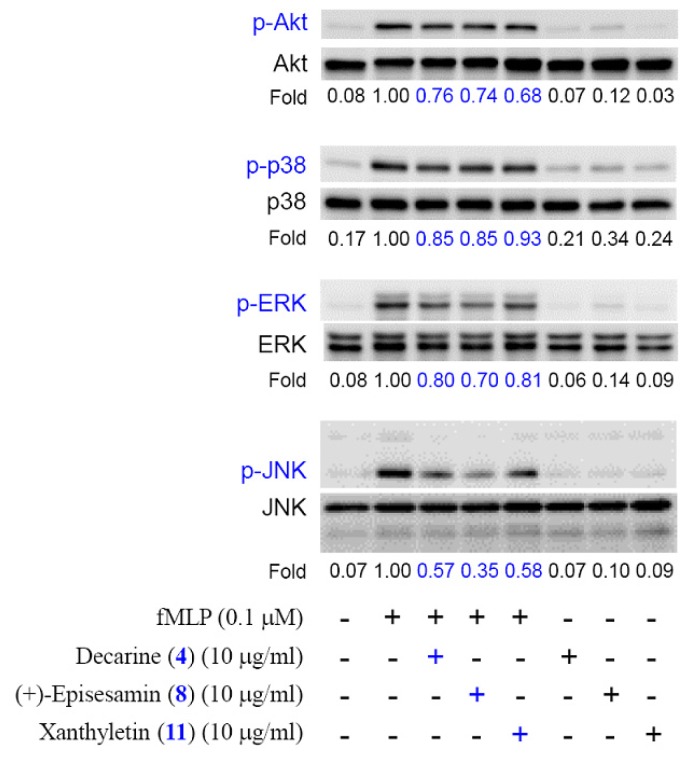
Compounds **4**, **8**, and **11** inhibit the phosphorylation of MAPKs and Akt in fMLP-activated neutrophils. Cells were treated with **4**, **8**, and **10** (10 μg/mL) for 5 min, and then stimulated with fMLP for 30 s. Phosphorylation of MAPKs and Akt was analyzed by immunoblotting. Densitometric analysis of all samples was normalized to the corresponding total protein.

The action mechanisms of **4**, **8**, and **11** in human neutrophils were further investigated. Mitogen-activated protein kinases (MAPKs) and phosphatidylinositol 3-kinase/Akt are the downstream signaling of fMLP in human neutrophils [31]. Compounds **4**, **8**, and **11** (10 μg/mL) caused a significant reduction of the phosphorylation of Akt and MAPks in fMLP-induced neutrophils (Figure 5). Notably, phosphorylation of JNK caused by fMLP was most significantly inhibited by these compounds. These results suggest that the anti-inflammatory effects of compounds **4**, **8**, and **11** are through the inhibition of activation of MAPKs and Akt in fMLP-activated neutrophils. Our study suggests *Z. ailanthoides* and its isolates (especially **4**, **8**, and **11**) could be further developed as potential candidates for the treatment or prevention of various inflammatory diseases.

## 3. Experimental Section

### 3.1. General Experimental Procedures

Melting points were determined on a Yanaco micro-melting point apparatus (Yanaco, Kyoto, Japan) and were uncorrected. Optical rotations were measured using a Jasco DIP-370 (Jasco, Tokyo, Japan) in CHCl_3_. Ultraviolet (UV) spectra were obtained on a Jasco UV-240 spectrophotometer (Jasco, Tokyo, Japan). Infrared (IR) spectra (neat or KBr) were recorded on a Perkin Elmer 2000 FT-IR spectrometer (Perkin Elmer, Norwalk, CT, USA). Nuclear magnetic resonance (NMR) spectra, including correlation spectroscopy (COSY), nuclear Overhauser effect spectrometry (NOESY), heteronuclear multiple-bond correlation (HMBC), and heteronuclear single-quantum coherence (HSQC) experiments, were acquired using a Varian Unity 400 or a Varian Inova 500 spectrometer operating (Varian Cary, Palo Alto, CA, USA) at 400 or 500 MHz (^1^H) and 100 or 125 MHz (^13^C), respectively, with chemical shifts given in ppm (δ) using tetramethylsilane (TMS) as an internal standard. Electrospray ionisation (ESI) and high-resolution electrospray ionization (HRESI)-mass spectra were recorded on a Bruker APEX II (Bruker, Bremen, Germany) or a VG Platform Electrospray ESI/MS mass spectrometer (Fison, Villeurbanne, France). Silica gel (70–230, 230–400 mesh, Merck, Darmstadt, Germany) was used for column chromatography (CC). Silica gel 60 F-254 (Merck, Darmstadt, Germany) was used for thin-layer chromatography (TLC) and preparative thin-layer chromatography (PTLC).

### 3.2. Plant Material

The twigs of *Z. ailanthoides* were collected from Hengchun, Pingtung County, Taiwan, in January 2009 and identified by Dr. J.-J. Chen. A voucher specimen (ZA 2009) was deposited in the Department of Pharmacy, Tajen University, Pingtung, Taiwan.

### 3.3. Extraction and Isolation

The dried twigs (1.3 kg) of *Z. ailanthoides* were pulverized and extracted with MeOH (3 × 10 L) for 3 days. The extract was concentrated under reduced pressure at 35 °C, and the residue (132 g) was partitioned between EtOAc and H_2_O (1:1) to provide the EtOAc-soluble fraction (fraction A; 46 g). The H_2_O-soluble fraction was further extracted with BuOH, and the BuOH-soluble part (fraction B; 43 g) and the H_2_O-soluble one (fraction C; 40 g) were separated. Fraction A (46 g) was purified by CC (2.2 kg of SiO_2_, 70–230 mesh; CH_2_Cl_2_/MeOH gradient) to afford 13 fractions: A1–A13. Fraction A1 (2.2 g) was subjected to CC (100 g of SiO_2_, 230–400 mesh; CH_2_Cl_2_/actone 20:1, 1.0 L-fractions) to give 9 subfractions: A1-1–A1-9. Fraction A1-5 (255 mg) was purified by MPLC (11.5 g of SiO_2_, 230–400 mesh, CHCl_3_/MeOH 20:1, 300 mL**-**fractions) to give 11 subfractions: A1-5-1–A1-5-11. Fraction A1-5-4 (25 mg) was further purified by preparative TLC (SiO_2_; *n*-hexane/EtOAc 6:1) to obtain **11** (5.7 mg). Fraction A1-5-5 (31 mg) was further purified by preparative TLC (SiO_2_; CHCl_3_/actone 30:1) to afford **8** (7.2 mg). Fraction A2 (3.0 g) was subjected to CC (142 g of SiO_2_, 230–400 mesh; CH_2_Cl_2_/MeOH 35:1, 1.0 L-fractions) to give 6 subfractions: A2-1–A2-6. Fraction A2-3 (125 mg) was further purified by preparative TLC (SiO_2_; hexane/acetone 5:2) to obtain **12** (5.5 mg). Fraction A3 (4.8 g) was purified by CC (225 g of SiO_2_, 230–400 mesh; *n*-hexane/acetone 3:2–0:1, 1.2 L-fractions) to give 12 subfractions: A3-1–A3-12. Fraction A3-3 (310 mg) was purified by MPLC (14.5 g of SiO_2_, 230–400 mesh; CHCl_3_/MeOH 50:1–0:1, 350 mL-fractions) to give 12 subfractions: A3-3-1–A3-3-12. Fraction A3-3-6 (29 mg) was further purified by preparative TLC (SiO_2_; CHCl_3_/MeOH 30:1) to yield **4** (9.3 mg). Fraction A3-7 (170 mg) was purified by MPLC (8.5 g of SiO_2_, 230–400 mesh, CHCl_3_/MeOH 40:1, 200 mL**-**fractions) to give 6 subfractions: A3-7-1–A3-7-6. Fraction A3-7-5 (32 mg) was further purified by preparative TLC (SiO_2_; hexane/EtOAc 1:1) to yield **10** (7.3 mg). Fraction A3-10 (360 mg) was further purified by CC (17 g of SiO_2_, 230–400 mesh; CHCl_3_/MeOH 20:1, 500 mL-fractions) to give 8 subfractions: A3-10-1–A3-10-8. Fraction A3-10-2 (55 mg) was further purified by preparative TLC (SiO_2_; CH_2_Cl_2_/acetone 10:1) to obtain **1** (4.2 mg), **6** (5.8 mg), and **9** (6.3 mg). Fraction A8 (3.1 g) was subjected to CC (132 g of SiO_2_, 230–400 mesh; CHCl_3_/MeOH 15:1–0:1, 400 mL-fractions) to afford 14 subfractions: A8-1–A8-14. Fraction A8-6 (220 mg) was further purified by CC (12 g of SiO_2_, 230–400 mesh; CHCl_3_/EtOAc 2:1–0:1, 250 mL-fractions) to give 9 subfractions: A8-6-1–A8-6-9. Fraction A8-6-3 (28 mg) further purified by preparative TLC (SiO_2_; CH_2_Cl_2_/acetone 3:1) to afford **7** (6.2 mg). Fraction A9 (3.4 g) was subjected to CC (144 g of SiO_2_, 230–400 mesh; CHCl_3_/MeOH 10:1–0:1, 300-mL fractions) to afford 12 subfractions: A9-1–A9-12. Fraction A9-7 (146 mg) was further purified by preparative TLC (SiO_2_; CH_2_Cl_2_/MeOH 20:1) to obtain **3** (3.2 mg). Fraction A9-8 (275 mg) was purified by MPLC (12.4 g of SiO_2_, 230–400 mesh, CHCl_3_/EtOAc 1:1–0:1, 180 mL**-**fractions) to give 10 subfractions: A9-8-1–A9-8-10. Fraction A9-8-4 (32 mg) was further purified by preparative TLC (SiO_2_; hexane/EtOAc 1:1) to yield **14** (8.3 mg). Fraction A10 (3.2 g) was subjected to CC (135 g of SiO_2_, 230–400 mesh; *n*-hexane/acetone 3:1, 500 mL-fractions) to afford 10 subfractions: A10-1–A10-10. Fraction A10-2 (310 mg) was purified by MPLC (13.5 g of SiO_2_, 230–400 mesh, *n*-hexane/EtOAc 5:1–0:1, 200-mL**-**fractions) to give 7 subfractions: A10-2-1–A10-2-7. Fraction A10-2-3 (46 mg) was further purified by preparative TLC (SiO_2_; CH_2_Cl_2_/EtOAc, 10:1) to obtain **13** (9.5 mg). Fraction A10-2-5 (42 mg) was further purified by preparative TLC (SiO_2_; CHCl_3_) to afford **5** (6.8 mg). Fraction A10-2-6 (38 mg) was further purified by preparative TLC (SiO_2_; CHCl_3_/MeOH 60:1) to yield **2** (5.1 mg).

#### 3.3.1. Oxynorchelerythrine (**1**)

White amorphous powder. UV (MeOH): λ_max_ (log ɛ) = 236 (4.89), 281 (3.61), 286 (4.65) nm. IR (KBr): υ_max_ = 3218 (NH), 1644 (C=O), 1040, 938 (OCH_2_O) cm^−1^. ^1^H-NMR (CDCl_3_, 500 MHz): δ = 4.01 (3H, s, OMe-8), 4.05 (3H, s, OMe-7), 6.13 (2H, s, OCH_2_O-2,3), 7.20 (1H, s, H-1), 7.43 (1H, br s, H-4), 7.46 (1H, d, *J* = 9.0 Hz, H-9), 7.51 (1H, br d, *J* = 9.0 Hz, H-12), 8.02 (1H, d, *J* = 9.0 Hz, H-11), 8.09 (1H, d, *J* = 9.0 Hz, H-10), 9.14 (1H, br s, D_2_O exchangeable, NH). ^13^C-NMR (CDCl_3_, 125 MHz): δ = 56.5 (OMe-8), 61.8 (OMe-7), 101.5 (OCH_2_O), 102.5 (C-4), 104.6 (C-1), 117.1 (C-4a), 117.7 (C-9), 117.8 (C-10), 118.4 (C-12), 119.7 (C-6a), 121.0 (C-10a), 123.3 (C-11), 128.9 (C-10b), 131.6 (C-12a), 135.6 (C-4b), 147.0 (C-3), 147.5 (C-2), 150.0 (C-7), 152.6 (C-8), 162.4 (C-6). ESI-MS: *m*/*z* = 372 [M + Na]^+^. HR-ESI-MS: *m*/*z* = 372.0846 [M + Na]^+^ (calcd for C_20_H_15_NO_5_Na: 372.0848).

#### 3.3.2. Methyl 4-(2-Hydroxy-4-methoxy-3-methyl-4-oxobutoxy)benzoate (**2**)

Colorless oil. UV (MeOH): λ_max_ (log ɛ) = 254 (3.96) nm. IR (neat): υ_max_ 3480 (OH), 1728 (C=O), 1714 (C=O) cm^−1^. ^1^H-NMR: see Table 1. ^13^C-NMR: see Table 1. ESI-MS: *m*/*z* = 305 [M + Na]^+^. HR-ESI-MS: *m*/*z* = 305.1003 [M + Na]^+^ (calcd for C_14_H_18_O_6_Na: 305.1001).

#### 3.3.3. (*E*)-Methyl 4-(4-((*Z*)-3-methoxy-3-oxoprop-1-enyl)phenoxy)-2-methylbut-2-enoate (**3**)

Amorphous powder. UV (MeOH): λ_max_ (log ɛ) = 296 (4.18) nm. IR (KBr): υ_max_ = 1715 (C=O) cm^−1^. ^1^H-NMR (CDCl_3_, 400 MHz): δ = 1.93 (3H, s, H-5′), 3.73 (3H, s, OMe-9), 3.76 (3H, s, OMe-4′), 4.69 (2H, d, *J* = 5.6 Hz, H-1′), 5.83 (1H, d, *J* = 12.4 Hz, H-8), 6.86 (1H, d, *J* = 12.4 Hz, H-7), 6.89 (2H, d, *J* = 8.8 Hz, H-3 and H-5), 6.93 (1H, br t, *J* = 5.6 Hz, H-2′), 7.69 (2H, d, *J* = 8.8 Hz, H-2 and H-6). ^13^C-NMR (CDCl_3_, 100 MHz): δ = 13.0 (C-5′), 51.3 (OMe-9), 51.5 (OMe-4′), 64.8 (C-1′), 114.2 (C-3), 114.2 (C-5), 116.7 (C-8), 127.3 (C-1), 129.6 (C-3′), 132.2 (C-2), 132.2 (C-6), 137.0 (C-2′), 143.6 (C-7), 159.2 (C-4), 166.9 (C-9), 167.8 (C-4′). ESI-MS: *m*/*z* = 313 [M + Na]^+^. HR-ESI-MS: *m*/*z* = 313.1055 [M + Na]^+^ (calcd for C_16_H_18_O_5_Na: 313.1052).

### 3.4. Biological Assay

The effect of the isolated compounds on neutrophil pro-inflammatory response was evaluated by monitoring the inhibition of superoxide anion generation and elastase release in fMLP/CB-activated human neutrophils in a concentration-dependent manner. The purity of the tested compounds was >98% as identified by NMR and MS. LY294002 (purity >99%, Sigma, St. Louis, MO, USA) was used as a positive control.

#### 3.4.1. Preparation of Human Neutrophils

Human neutrophils from venous blood of healthy, adult volunteers (20–28 years old) were isolated using a standard method of dextran sedimentation prior to centrifugation in a Ficoll Hypaque gradient and hypotonic lysis of erythrocytes [32]. Purified neutrophils containing >98% viable cells, as determined by the trypan blue exclusion method [33], were re-suspended in a calcium (Ca^2+^)-free HBSS buffer at pH 7.4 and were maintained at 4 °C prior to use.

#### 3.4.2. Measurement of Superoxide Anion Generation

The assay for measurement of superoxide anion generation was based on the SOD-inhibitable reduction of ferricytochrome *c* [34,35]. In brief, after supplementation with 0.5 mg/mL ferricytochrome *c* and 1 mM Ca^2+^, neutrophils (6 × 10^5^/mL) were equilibrated at 37 °C for 2 min and incubated with different concentrations (10–0.01 μg/mL) of compounds or DMSO (as control) for 5 min. Cells were incubated with cytochalasin B (1 μg/mL) for 3 min prior to the activation with 100 nM formyl-l-methionyl-l-leucyl-l-phenylalanine for 10 min. Changes in absorbance with the reduction of ferricytochrome *c* at 550 nm were continuously monitored in a double-beam, six-cell positioner spectrophotometer with constant stirring (Hitachi U-3010, Tokyo, Japan). Calculations were based on differences in the reactions with and without SOD (100 U/mL) divided by the extinction coefficient for the reduction of ferricytochrome *c* (*ɛ* = 21.1/mM/10 mm).

#### 3.4.3. Measurement of Elastase Release

Degranulation of azurophilic granules was determined by measuring elastase release as described previously [35]. Experiments were performed using MeO-Suc-Ala-Ala-Pro-Val-*p*-nitroanilide as the elastase substrate. Briefly, after supplementation with MeO-Suc-Ala-Ala-Pro-Val-*p*-nitroanilide (100 μM), neutrophils (6 × 10^5^/mL) were equilibrated at 37 °C for 2 min and incubated with compounds for 5 min. Cells were stimulated with fMLP (100 nM)/CB (0.5 μg/mL), and changes in absorbance at 405 nm were monitored continuously in order to assay elastase release. The results were expressed as the percent of elastase release in the fMLP/CB-activated, drug-free control system.

#### 3.4.4. Western Analysis

Neutrophils were preincubated with compounds for 5 min before adding fMLP at 37 °C for 30 s. Cells was lysed in 5 × Laemmli’s sample buffer. Cell lysates were subjected to Immunoblotting, and the immunoreactive bands were visualized by an enhanced chemiluminescence system (Amersham Biosciences, Foster City, CA, USA) and detected by UVP imaging system (UVP, Upland, CA, USA) [36,37].

#### 3.4.5. Statistical Analysis

Results are expressed as the mean ± SEM, and comparisons were made using Student’s *t*-test. A probability of 0.05 or less was considered significant. The software SigmaPlot (Systat Software, San Jose, CA, USA) was used for the statistical analysis.

## 4. Conclusions

Fourteen compounds, including a new benzo[*c*]phenanthridine (**1**) and two new benzenoids (**2** and **3**), were isolated from the twigs of *Z. ailanthoides*. The structures of these compounds were established on the basis of spectroscopic data. Reactive oxygen species (ROS) [e.g., superoxide anion (O_2_^•−^), hydrogen peroxide] and granule proteases (e.g., elastase, cathepsin G) produced by human neutrophils contribute to the pathogenesis of inflammatory diseases. The effects on neutrophil pro-inflammatory responses of isolates were evaluated by suppressing fMLP/CB-induced O_2_^•−^ generation and elastase release by human neutrophils. The results of anti-inflammatory experiments indicate that compounds **4**, **6**, and **8**–**11** can significantly inhibit fMLP-induced O_2_^•−^ generation and/or elastase release. Decarine (**4**) and (+)-episesamin (**8**) were the most effective among the isolated compounds, with IC_50_ values of 1.31 ± 0.18 and 1.42 ± 0.16 μg/mL, respectively, against fMLP-induced O_2_^•−^ generation and elastase release. Compounds **4**, **8**, and **11** (10 μg/mL) caused a significant reduction of the phosphorylation of Akt and MAPks in fMLP-induced neutrophils. Thus, the anti-inflammatory effects of compounds **4**, **8**, and **11** are through the inhibition of activation of MAPKs and Akt in fMLP-activated neutrophils. Our study suggests *Z. ailanthoides* and its isolates (especially **4**, **8**, and **11**) could be further developed as potential candidates for the treatment or prevention of various inflammatory diseases.

## Supplementary Information



## Figures and Tables

**Table 1:** ^1^H- and ^13^C-NMR data of **2**. At 500 (^1^H) and 125 MHz (^13^C) in CDCl_3_; δ in ppm, *J* in Hz.

Position	δ_C_	δ_H_	NOESY	HMBC a
1	123.1			
2	131.6	7.99 (d, *J* = 9.0)	3, MeOCO-1	3, 4, 6, MeO*C*O-1
3	114.1	6.93 (d, *J* = 9.0)	2, 1′	1, 4, 5
4	162.1			
5	114.1	6.93 (d, *J* = 9.0)	6, 1′	1, 3, 4
6	131.6	7.99 (d, *J* = 9.0)	5, MeOCO-1	2, 4, 5, MeO*C*O-1
1′	69.8	4.11 (m)	3, 5, 3′, 5′	4, 2′, 3′
2′	71.8	4.11 (m)	3′, 5′, OH-2′	1′, 4′, 5′
3′	42.0	2.88 (m)	2′, 5′	1′, 2′, 4′
4′	175.7			
5′	14.1	1.30 (d, *J* = 7.0)	1′, 2′, 3′, OMe-4′	2′, 3′, 4′
*Me*OCO-1	51.9	3.89 (s)	2, 6	MeO*C*O-1
MeO*C*O-1	166.7			
OH-2′		3.09 (br s)	2′	
OMe-4′	52.0	3.74 (s)	5′	4′

a From the H- to the C-atom.

**Table 2:** Inhibitory effects of compounds **1**–**14** from the twigs of *Zanthoxylum ailanthoides* on superoxide radical anion generation and elastase release by human neutrophils in response to fMet-Leu-Phe/cytochalasin B a.

Compounds	Superoxide anion	Elastase

	IC_50_ [μg/mL] b or (Inh %) c	
Oxynorchelerythrine (**1**)	(29.69 ± 1.29) g	(20.28 ± 5.20) f
Methyl 4-(2-hydroxy-4-methoxy-3-methyl-4-oxobutoxy)benzoate (**2**)	(19.46 ± 4.19) f	(8.32 ± 2.49) e
(*E*)-methyl 4-(4-((Z)-3-methoxy-3-oxoprop-1-enyl)phenoxy)-2-methylbut-2-enoate (**3**)	(33.42 ± 4.53) f	(24.15 ± 3.22) e
Decarine (**4**)	1.31 ± 0.18 g	1.95 ± 0.28 g
6-Acetonyldihydrochelerythrine (**5**)	(48.36 ± 4.85) f	7.12 ± 0.31 e
(−)-Syringaresinol (**6**)	4.79 ± 0.39 g	(7.66 ± 3.71)
5′,5″-Didemethoxypinoresinol (**7**)	(45.22 ± 3.31) g	(23.91 ± 5.75) e
(+)-Episesamin (**8**)	4.33 ± 0.56 g	1.42 ± 0.16 g
Glaberide I (**9**)	3.98 ± 0.44 g	(23.00 ± 2.92) f
(−)-Dihydrocubebin (**10**)	2.42 ± 0.47 f	(32.78 ± 4.94) f
Xanthyletin (**11**)	4.16 ± 0.35 g	5.48 ± 0.27 g
Lanyulactone (**12**)	(36.03 ± 5.00) f	(34.55 ± 6.14) f
Methyl 3,4-dimethoxybenzoate (**13**)	(40.21 ± 6.27) e	(29.96 ± 6.18) e
*p*-Hydroxybenzoic acid (**14**)	(17.30 ± 9.77) g	(32.79 ± 1.48) g
LY294002 d	1.14 ± 0.12 f	1.94 ± 0.23 f

a Results are presented as averages ± SEM (*n* = 4);

b Concentration necessary for 50% inhibition (IC_50_);

c Percentage of inhibition (Inh%) at 10 μg/mL;

d LY294002, a phosphatidylinositol-3-kinase inhibitor, was used as a positive control for superoxide anion generation and elastase release;

e *p* < 0.05 compared with the control;

f *p* < 0.01 compared with the control;

g *p* < 0.001 compared with the control.
