# Supplementary material for: New Benzo[c]phenanthridine and Benzenoid Derivatives, and Other Constituents from Zanthoxylum ailanthoides: Effects on Neutrophil Pro-Inflammatory Responses
Source: Int J Mol Sci. 2013 Nov 13;14(11):22395–408. doi: 10.3390/ijms141122395 (PMC3856070; doi:10.3390/ijms141122395)

Supplementary Information

Figure S1. ESI-MS spectrum of **1**.

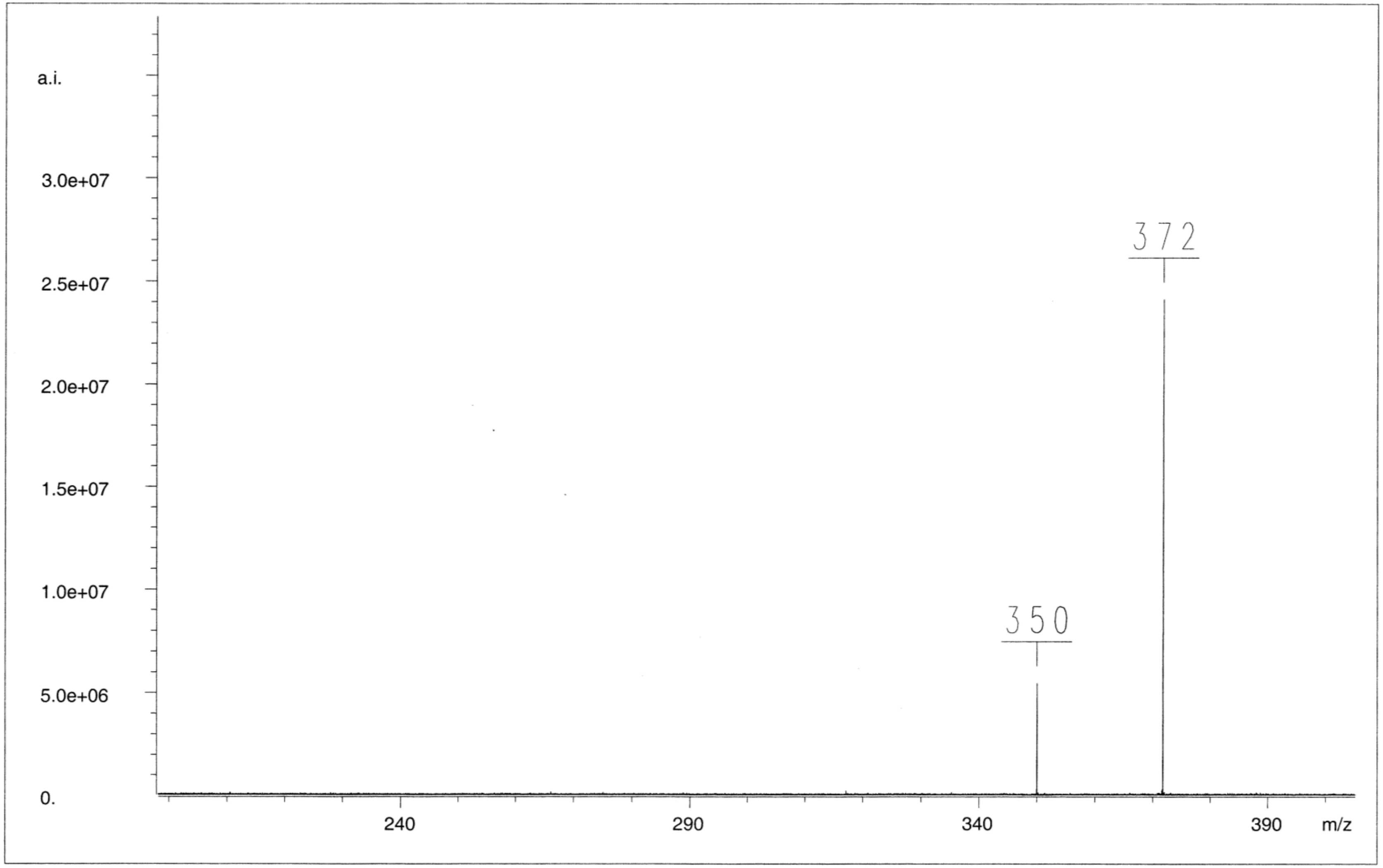

Figure S2. HR-ESI-MS spectrum of **1**.

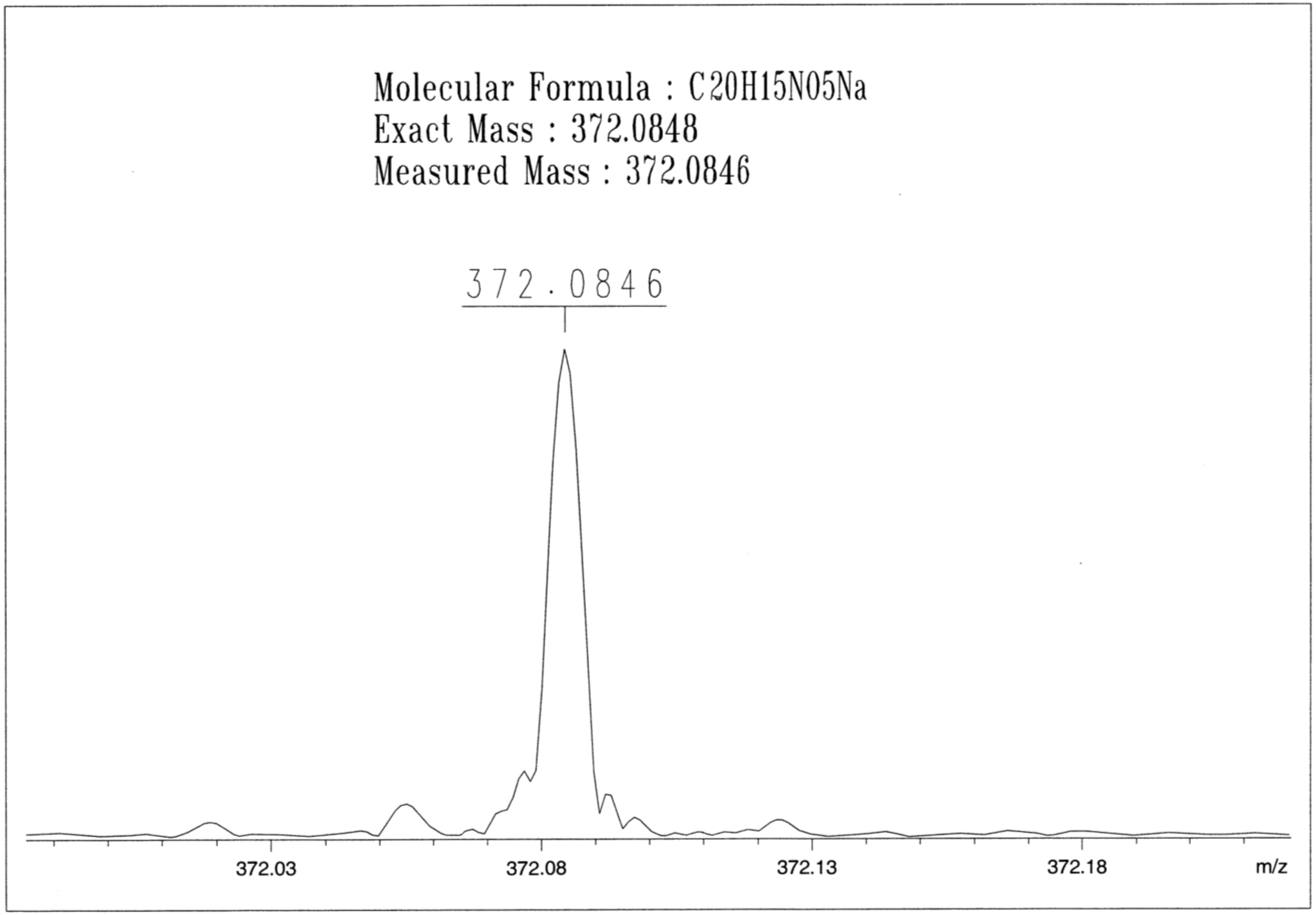

Figure S3. ^{1}H-NMR spectrum ($CDCl_3$, 400 MHz) of **1**.

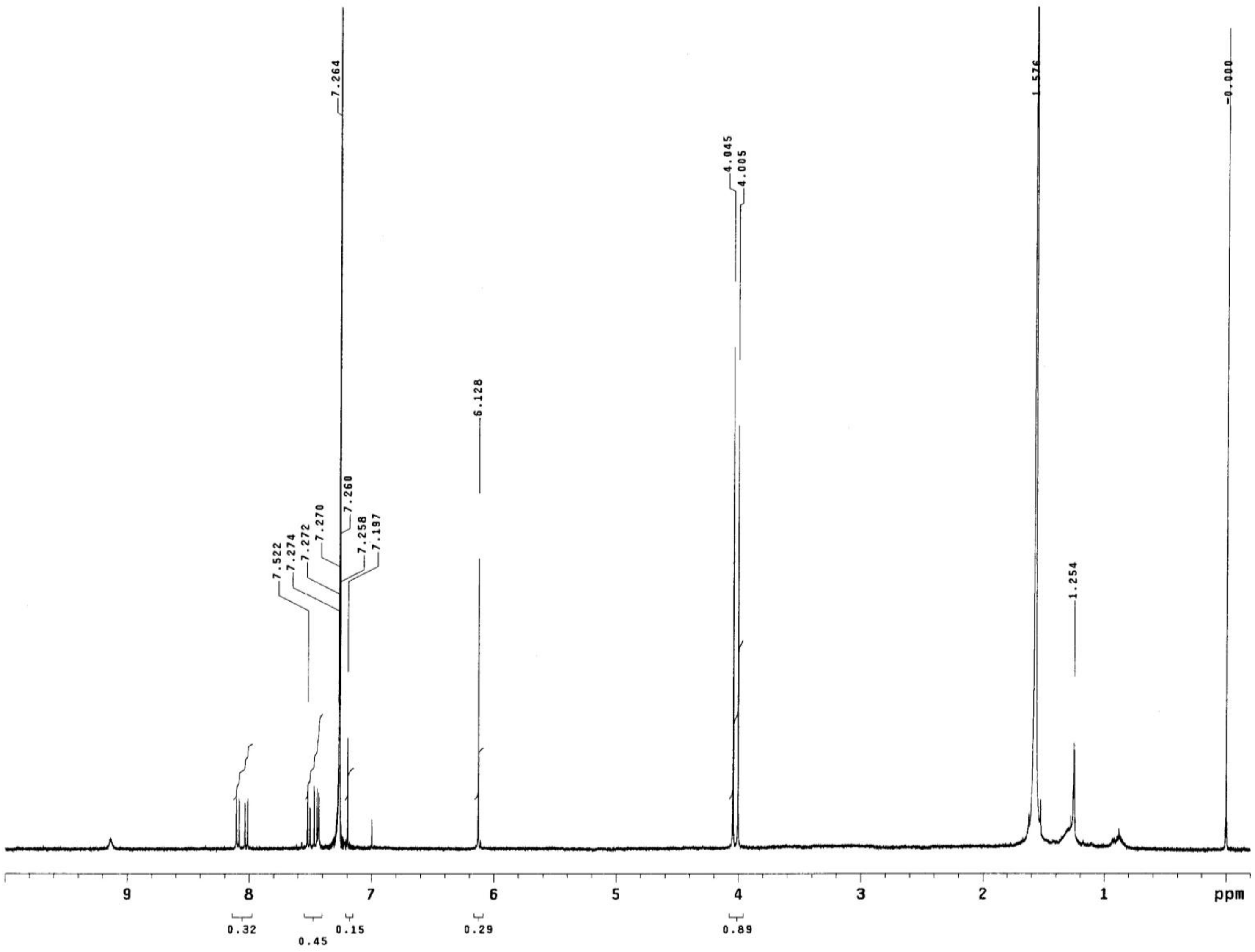

Figure S4. ^{13}C-NMR spectrum ($CDCl_3$, 100 MHz) of **1**.

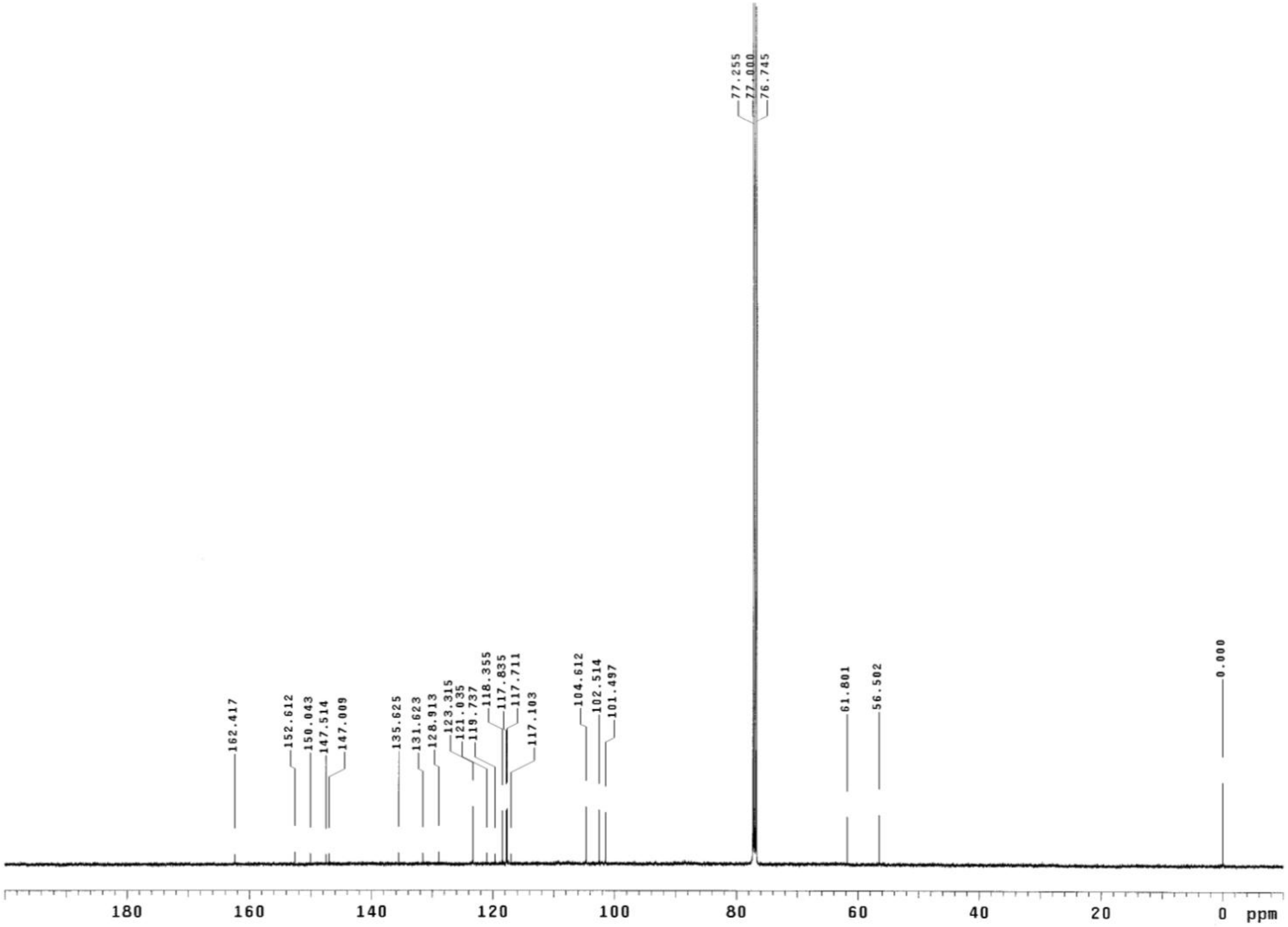

Figure S5. ESI-MS spectrum of **2**.

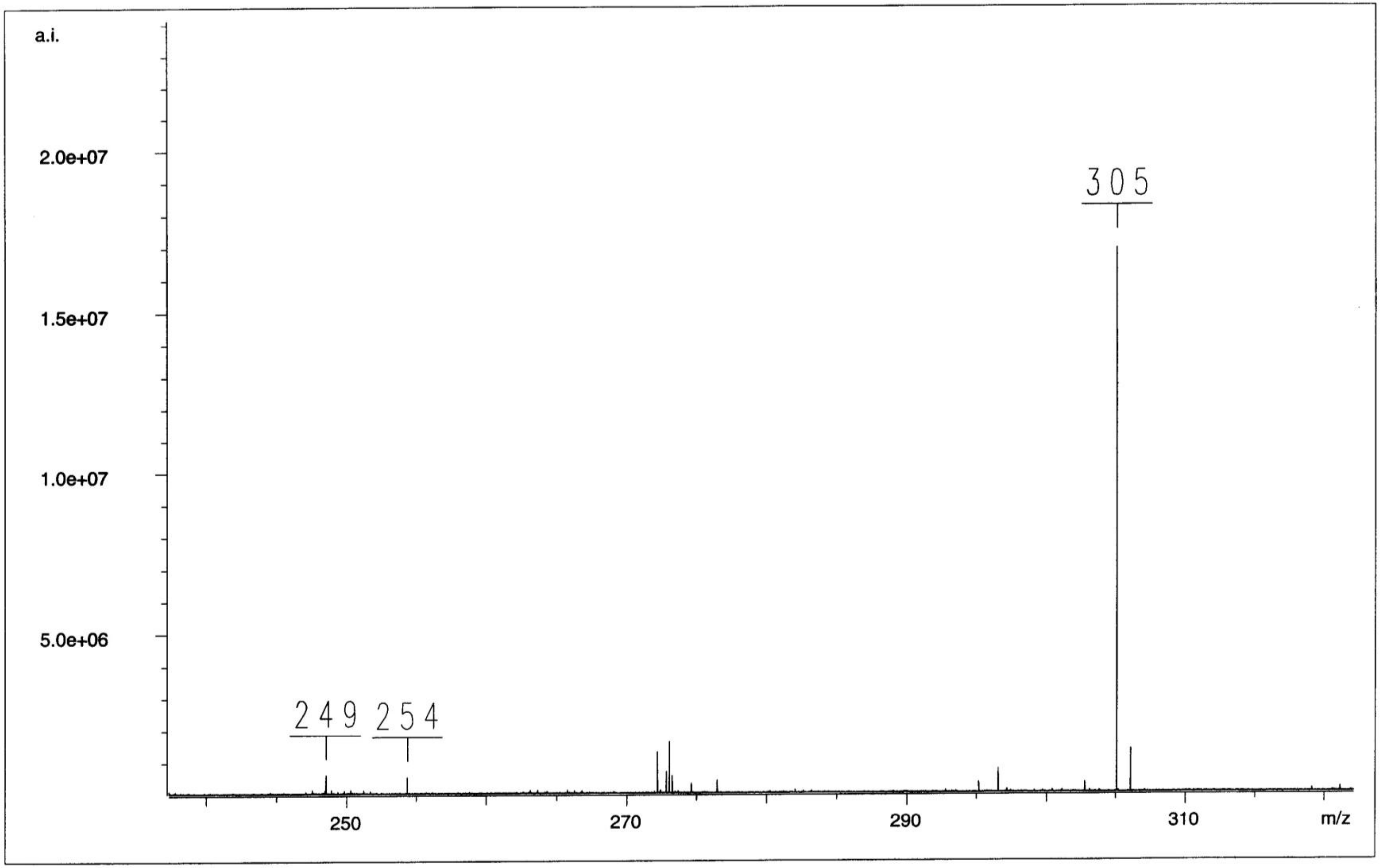

Figure S6. HR-ESI-MS spectrum of **2**.

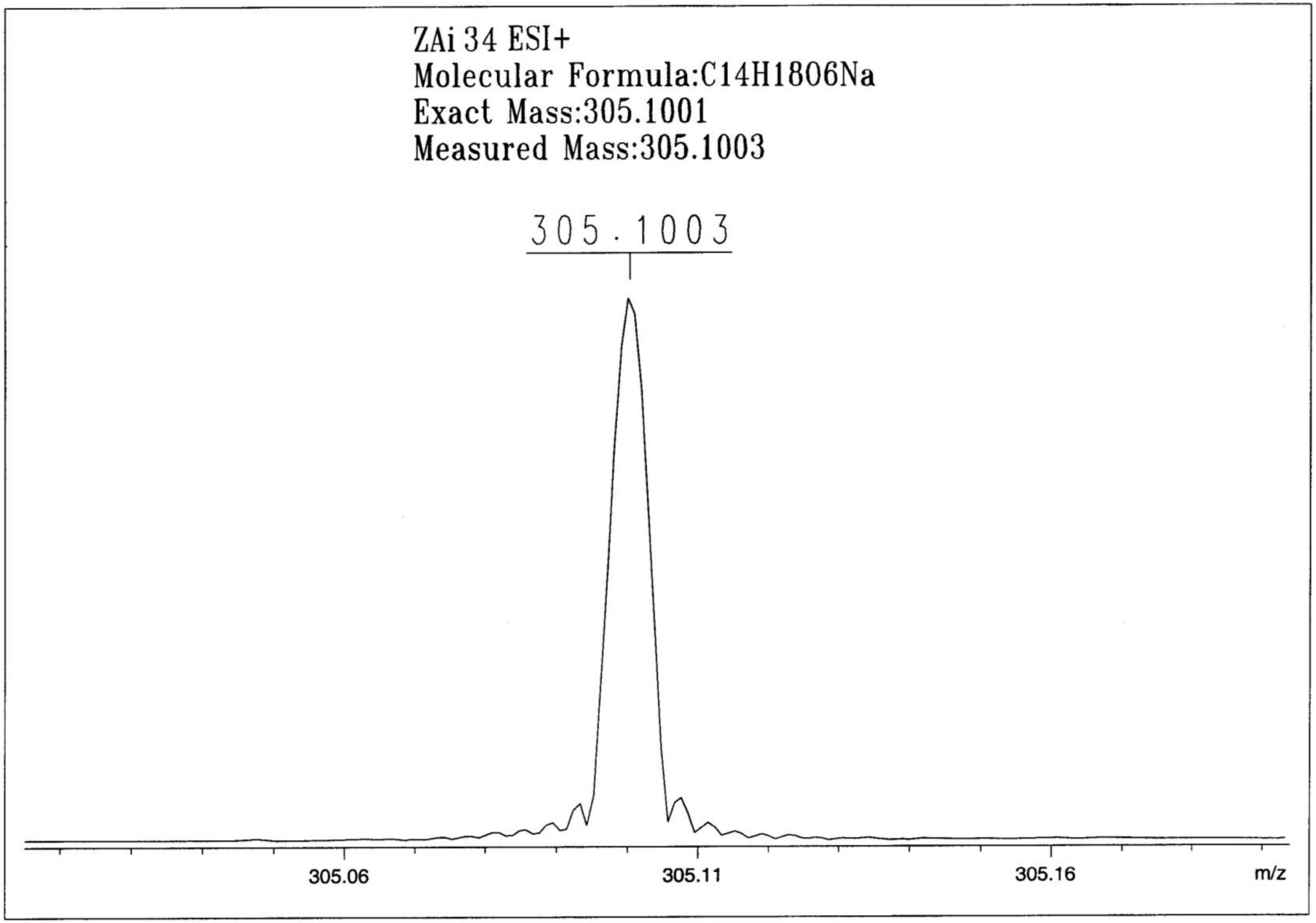

Figure S7. ^{1}H-NMR spectrum ($CDCl_3$, 500 MHz) of **2**.

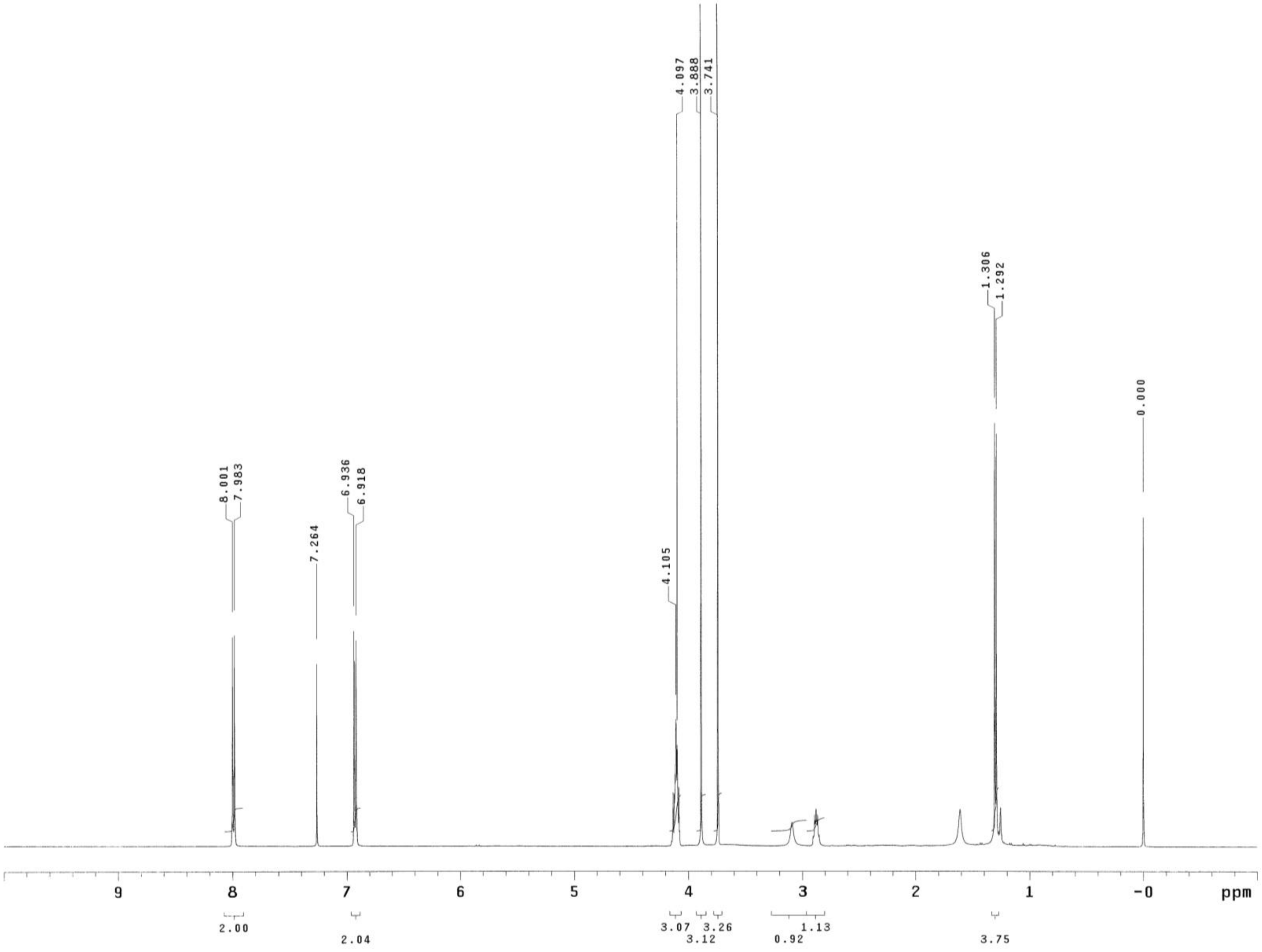

Figure S8. ^{13}C-NMR spectrum ($CDCl_3$, 125 MHz) of **2**.

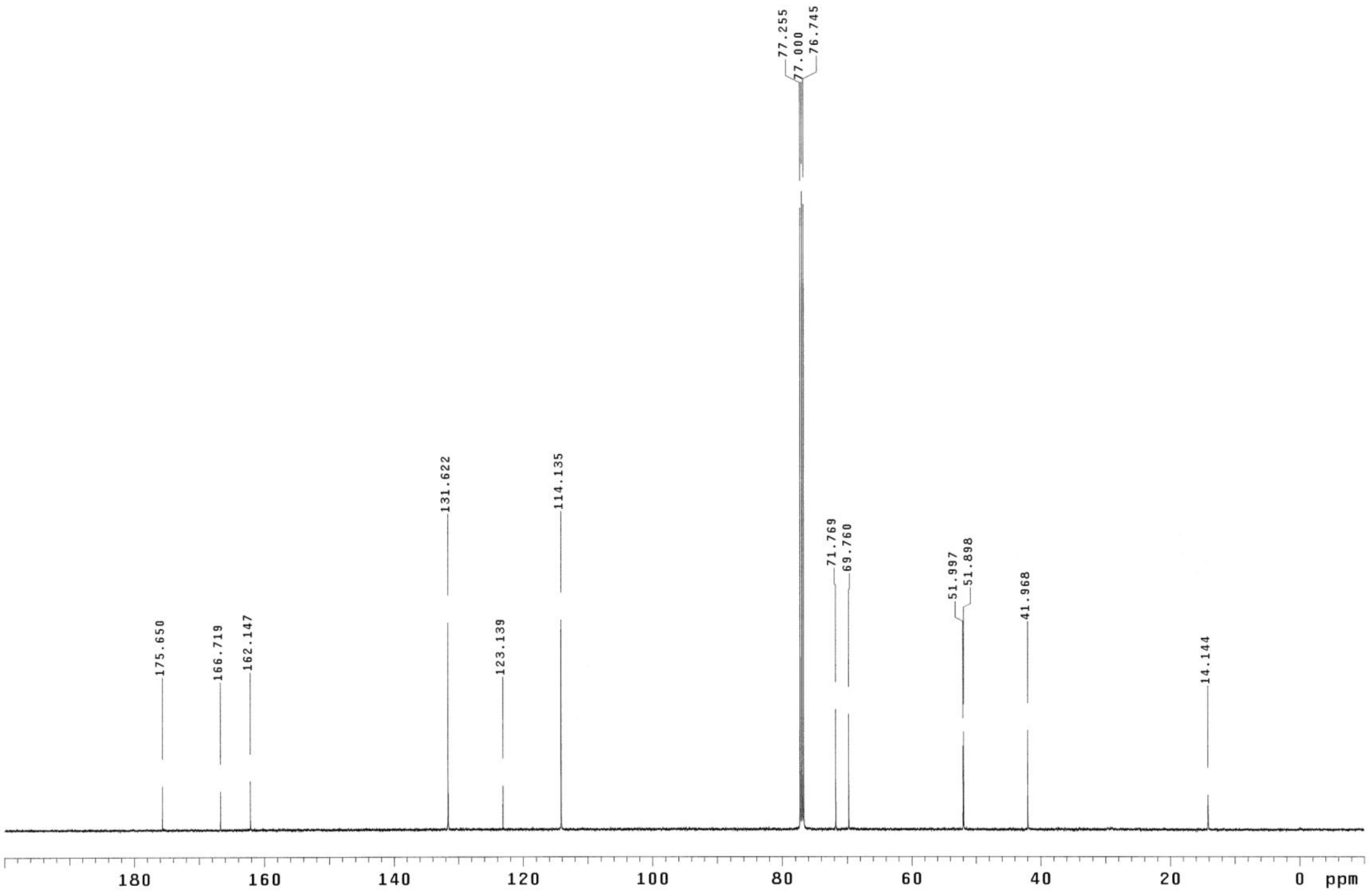

Figure S9. ESI-MS spectrum of **3**.

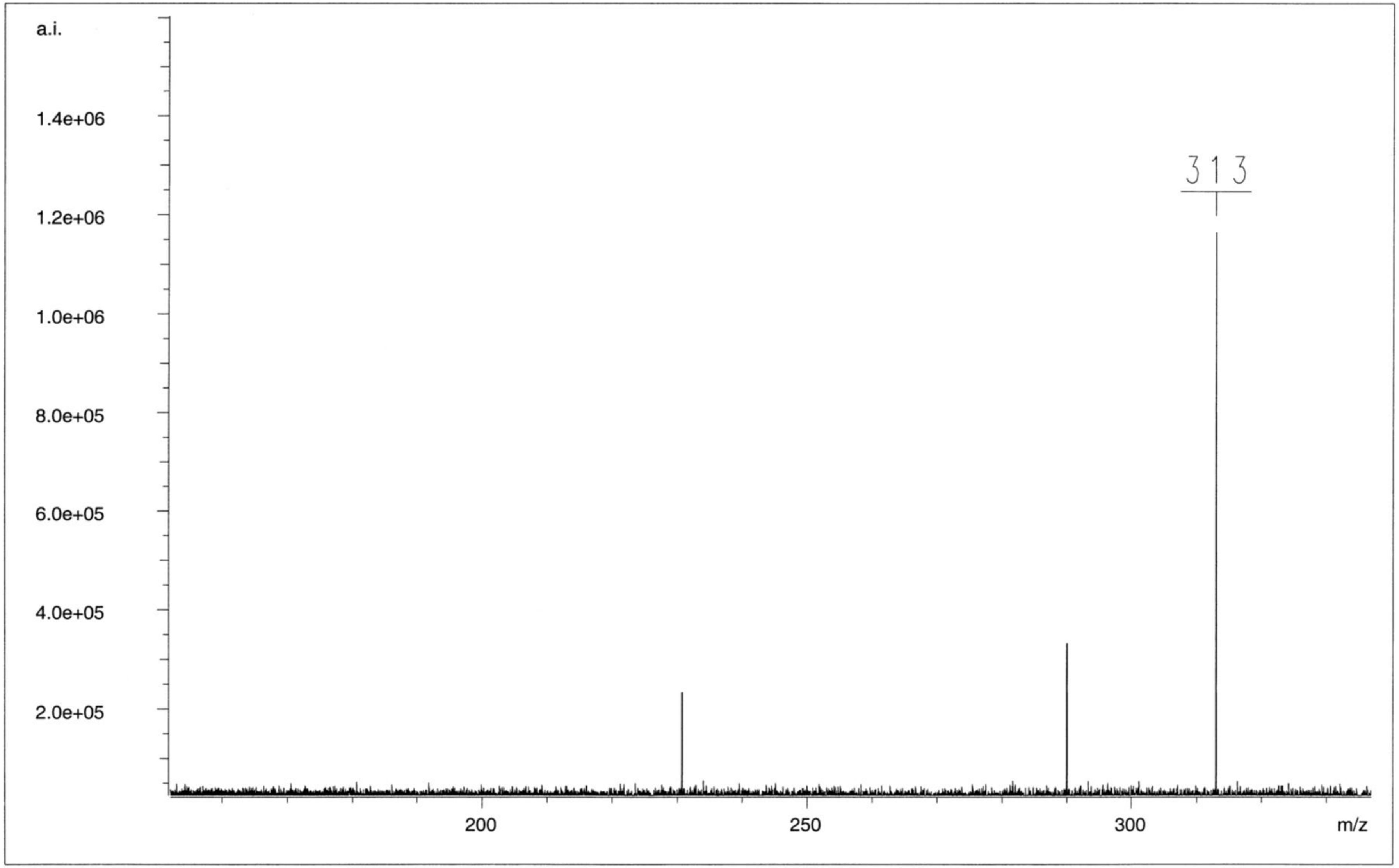

Figure S10. HR-ESI-MS spectrum of **3**.

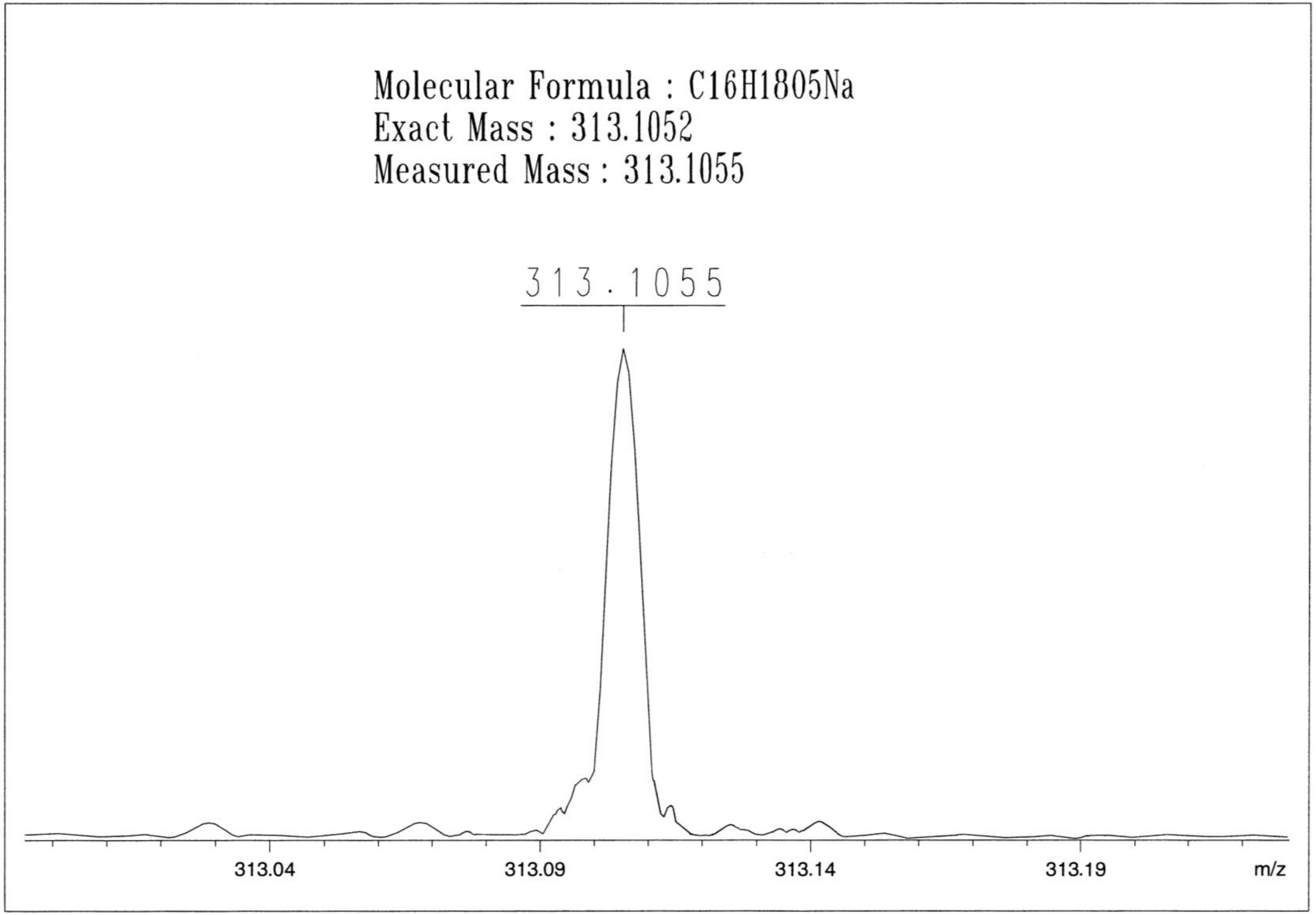

Figure S11. ^{1}H-NMR spectrum ($CDCl_3$, 400 MHz) of **3**.

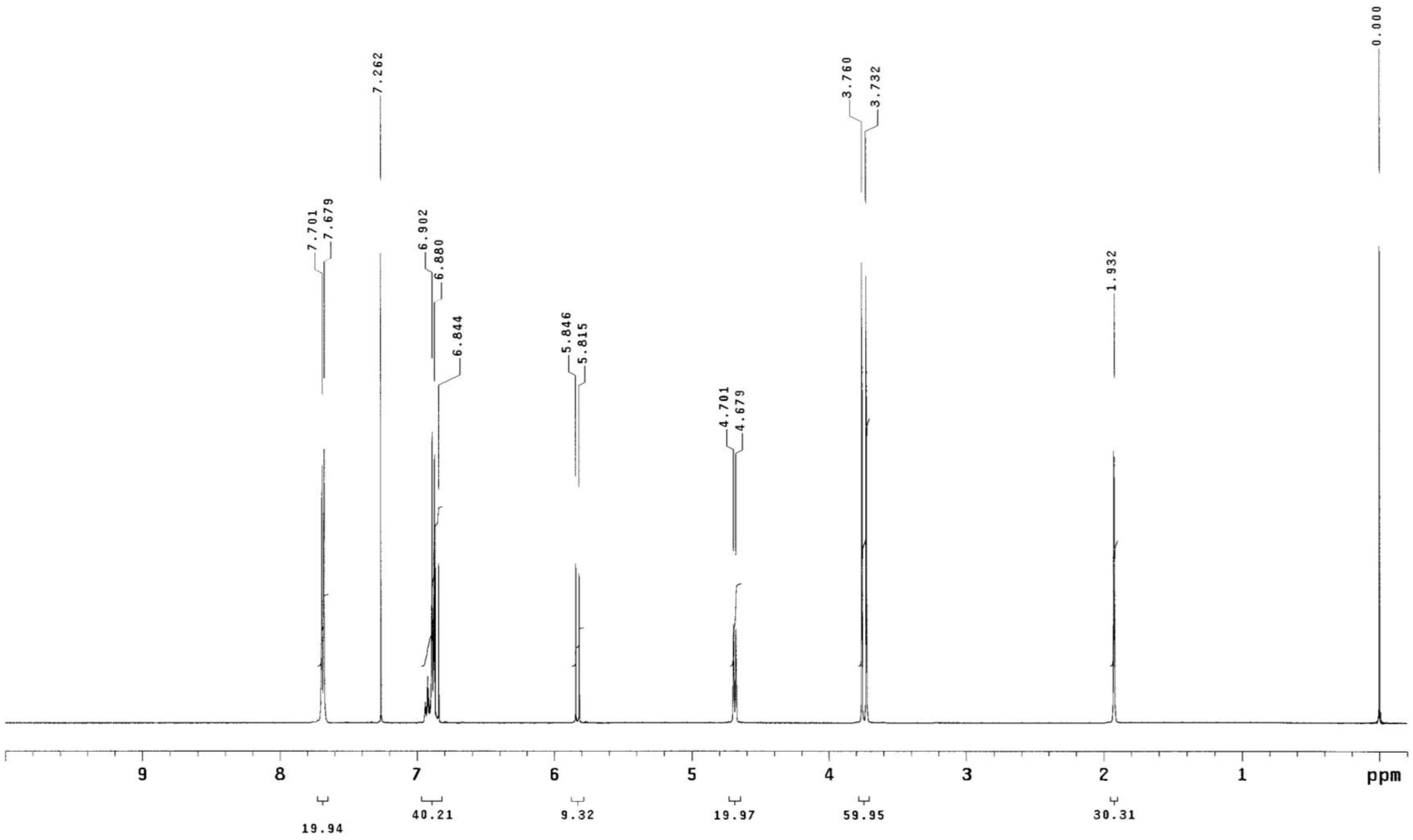

Figure S12. ^{13}C-NMR spectrum of **3** ($CDCl_3$, 100 MHz).

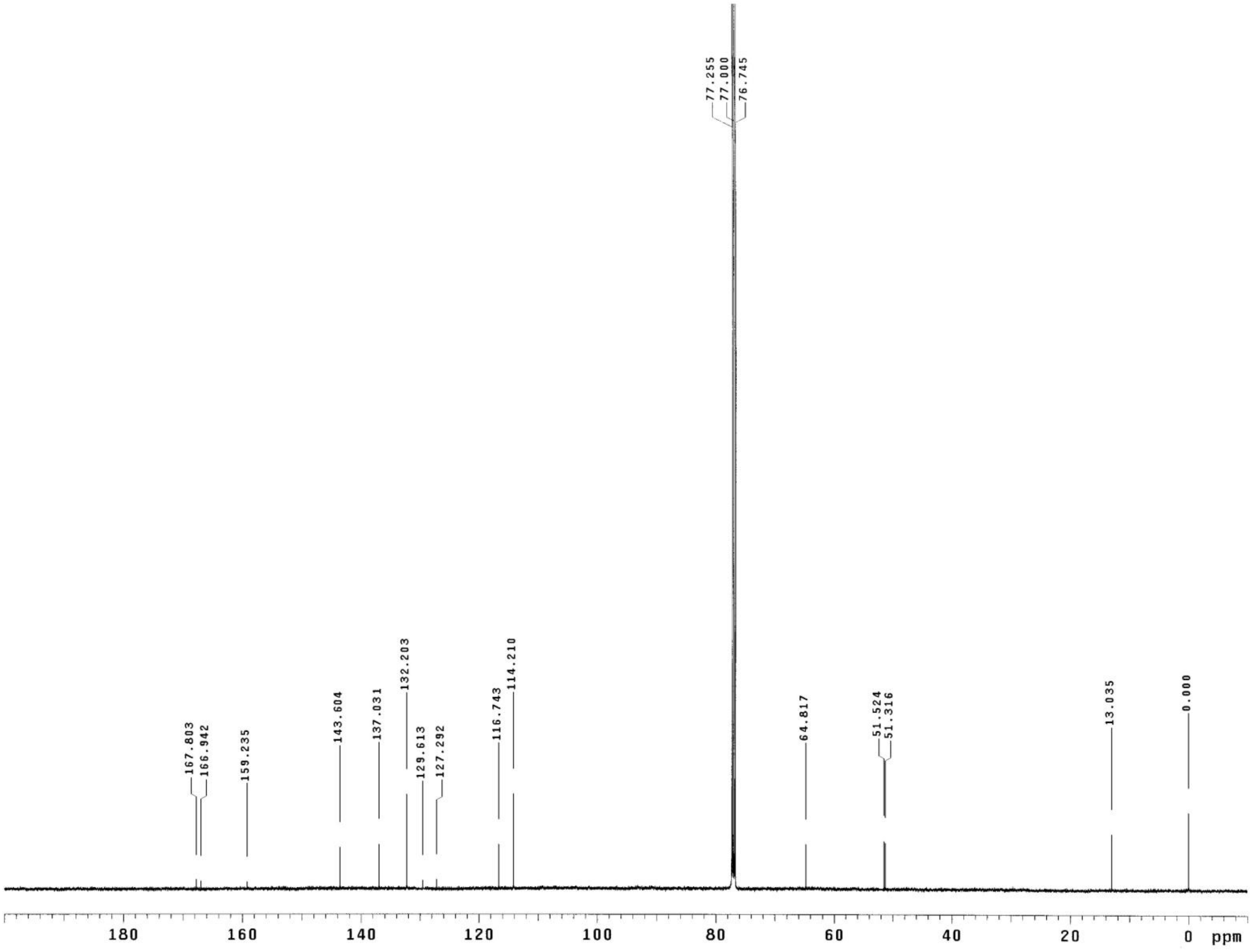

Supplement: Supplementary file 1 [file ijms-14-22395-s001.pdf]
